# Anti-CRISPR-mediated control of gene editing and synthetic circuits in eukaryotic cells

**DOI:** 10.1038/s41467-018-08158-x

**Published:** 2019-01-14

**Authors:** Muneaki Nakamura, Prashanth Srinivasan, Michael Chavez, Matthew A. Carter, Antonia A. Dominguez, Marie La Russa, Matthew B. Lau, Timothy R. Abbott, Xiaoshu Xu, Dehua Zhao, Yuchen Gao, Nathan H. Kipniss, Christina D. Smolke, Joseph Bondy-Denomy, Lei S. Qi

**Affiliations:** 1 Department of Bioengineering, Stanford University, Stanford, CA 94305 USA; 2 Department of Chemical and Systems Biology, Stanford University, Stanford, CA 94305 USA; 3 Stanford ChEM-H, Stanford University, Stanford, CA 94305 USA; 4 International Christian School, 1 On Muk Ln, Sha Tin, 999077 Hong Kong SAR China; 5 Cancer Biology Program, Stanford University, Stanford, CA 94305 USA; 6 Department of Microbiology and Immunology, University of California, San Francisco, CA 94158 USA; 7 Quantitative Biosciences Institute, University of California, San Francisco, CA 94158 USA

## Abstract

Repurposed CRISPR-Cas molecules provide a useful tool set for broad applications of genomic editing and regulation of gene expression in prokaryotes and eukaryotes. Recent discovery of phage-derived proteins, anti-CRISPRs, which serve to abrogate natural CRISPR anti-phage activity, potentially expands the ability to build synthetic CRISPR-mediated circuits. Here, we characterize a panel of anti-CRISPR molecules for expanded applications to counteract CRISPR-mediated gene activation and repression of reporter and endogenous genes in various cell types. We demonstrate that cells pre-engineered with anti-CRISPR molecules become resistant to gene editing, thus providing a means to generate “write-protected” cells that prevent future gene editing. We further show that anti-CRISPRs can be used to control CRISPR-based gene regulation circuits, including implementation of a pulse generator circuit in mammalian cells. Our work suggests that anti-CRISPR proteins should serve as widely applicable tools for synthetic systems regulating the behavior of eukaryotic cells.

## Abstract (web summary)

Anti-CRISPR proteins derived from phage can abrogate CRISPR activity. The authors repurpose these molecules for demonstrating genomic write-protection and pre-programmed gene expression circuits.

## Introduction

CRISPR systems, a form of prokaryotic adaptive immunity, have been widely repurposed for biotechnological applications, including genome editing and gene expression regulation in prokaryotic and eukaryotic organisms^1,2^. While these technological advances bring potential benefits to medicine, agriculture, and the environment, concomitant concerns arise over the largely irreversible outcomes generated by genome editing. Means to safely and reversibly control the activity of CRISPR tools can mitigate security concerns related to their accidental or intentional misuse. However, the tools available to effectively counteract gene editing or gene regulation remain limited.

Synthetic circuits controlling gene expression are another area of active interest for programming novel cellular behaviors, such as controlling development or implementing biological sensors and devices^3,4^. Recently, interest in synthetic circuits implemented by CRISPR systems^5,6^ has grown due to the adaptability of CRISPR-based gene regulation. The complexity of implementable circuits is limited by the types of control nodes that can be wired to control CRISPR systems. To this end, methods of exogenous control over CRISPR systems have been developed^7–12^, but internally programmed methods of control remain sparse.

The discovery of a set of bacteriophage proteins, anti-CRISPRs (Acrs), that inactivate certain CRISPR systems revealed the existence of an evolutionary arms race between these adaptive immune systems and infectious agents^13^; however, it was not until fairly recently that Acrs targeting the Class II CRISPR systems, including the protein mostly widely used for genomic engineering, *S. pyogenes* (*Spy*) CRISPR-associated protein 9 (Cas9), were identified^14,15^. Based on these reports and biophysical and biochemical analyses^16–18^, a picture has emerged by which these new Acrs can inhibit CRISPR activity by a variety of mechanisms and with varying promiscuity, but predominantly specifically inhibit the binding of a small set of Cas proteins to DNA. These studies demonstrated inhibition of gene expression in *E. coli* cells or extracts^15,19^, as well as inhibition of genomic editing^14,15,17,20^, imaging^14,20^, or deposition of epigenetic marks^20,21^. However, the broad extent as to whether Acrs can be used as tools to provide temporal, inhibitory control of CRISPR genome editors and nuclease-deactivated Cas9 (dCas9) genome regulators (both activation and repression) in different eukaryotic cells remains to be characterized. In this work, we present a more complete characterization of Acr activity in a range of contexts and establish the basis for biotechnological applications involving the use of Acrs for controlling CRISPR activity in mammalian cells.

## Results

### Acrs inhibit CRISPR-based gene regulation in mammalian cells

**Fig. 1:**
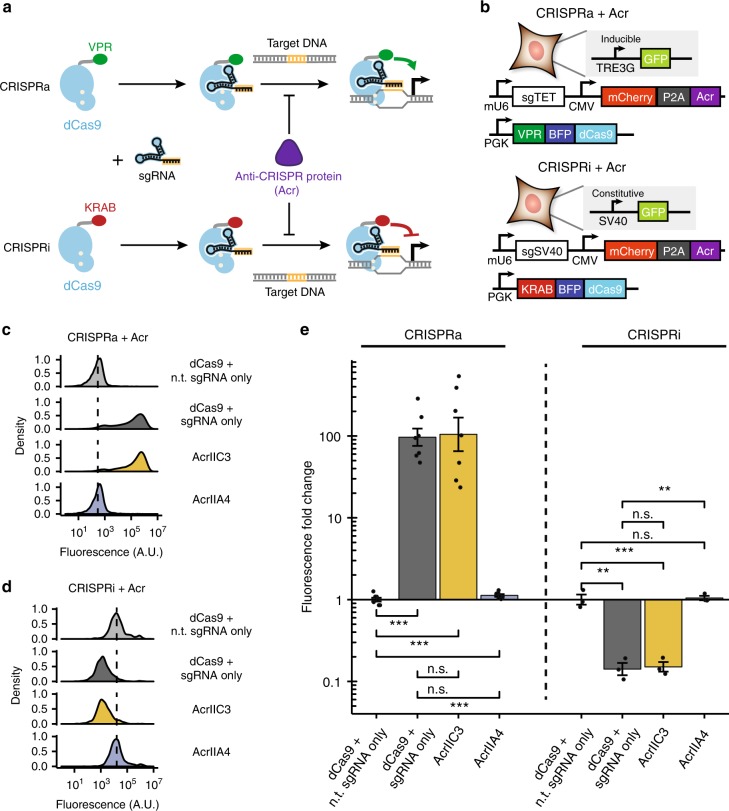
CRISPR-based gene regulation provides quantitative characterization of Acr activity. **a** Gene regulation mediated by sgRNA-programmed binding of dCas9 fused with gene activation domain VPR (CRISPRa) and gene repression domain KRAB (CRISPRi). Acrs that inhibit binding of DNA should prevent CRISPRa and CRISPRi. **b** Box diagrams for experimental assay of Acr activity: cell lines with integrated reporter for CRISPRa (top) and CRISPRi (bottom) are transiently transfected with plasmids encoding appropriate dCas9 effector, sgRNA, and Acr variants fused via P2A peptide to mCherry. **c**, **d** Representative raw fluorescence flow cytometry traces for CRISPRa (**c**) and CRISPRi (**d**) assays. Negative control (dCas9 effector + non-targeting [n.t.] sgRNA) and positive control (dCas9 effector + active sgRNA) conditions in absence of Acr are compared to conditions with AcrIIC3 or AcrIIA4 incorporated in active sgRNA plasmid. Dotted line indicates median value of non-targeting sgRNA negative control. **e** Summary comparison of CRISPRa and CRISPRi activity in presence and absence of active and null Acrs (n.s.: *p* > 0.05; **: *p* < 0.01; ***: *p* < 0.001) for *n* = 7 (CRISPRa) and *n* = 3 (CRISPRi) experimental replicates. Source Data are provided as a Source Data file. Error bars indicate ± s.e.m.

CRISPR-based regulation of gene expression involves the use of a dCas9 with target sequence specified by a single guide RNA (sgRNA). While DNA binding of dCas9 alone is sufficient for CRISPR-based gene interference (CRISPRi) in prokaryotes^22–24^ and yeast^25,26^, optimal CRISPRi or CRISPR-based gene activation (CRISPRa) in most eukaryotic organisms involves the fusion of repressive or activating domains to dCas9, which, when targeted to a particular locus by an sgRNA, results in specific gene down- or upregulation^25,27^. We therefore hypothesized that Acrs that function through the inhibition of Cas9 binding to DNA should be able to inhibit CRISPRa and CRISPRi (Fig. 1a), and, conversely, we could use these gene regulation tools to further characterize the function of Acrs.

To do this, we systematically assessed the efficacy of a panel of 5 Acrs (AcrIIC1, AcrIIA1, AcrIIA2, AcrIIA3, AcrIIA4) targeting Class II CRISPR systems^14,15^ to alter gene expression changes induced by CRISPRa and CRISPRi. We co-transfected plasmids encoding dCas9-effectors, sgRNA, and Acrs into suitable HEK293T reporter cell lines (Supplementary Fig. 1a). For CRISPRa, we transfected VPR-dCas9 into a reporter line bearing the inducible TRE3G promoter driving GFP expression; for CRISPRi, we used KRAB-dCas9 on a line with an SV40 promoter driving GFP. For both cell lines, the sgRNA sequence was designed to target the region of the promoter proximal to the transcription start site and cells were assessed for induced GFP reporter expression change.

We observed varying levels of inhibition of CRISPRa and CRISPRi caused by these Acrs, with AcrIIA4 demonstrating consistently significant effect in negating CRISPR gene regulation (Supplementary Fig. 1b-c), which is generally consistent with results involving CRISPRi in bacteria and editing in human cells^15^, as well as the proposed mechanism of AcrIIA4 inhibiting DNA binding by serving as a DNA mimic^16,17^.

Based on these results, we further explored the utility of the best-performing AcrIIA4 in controlling gene regulation. We designed an optimized version of our assay, wherein the sgRNA and Acr were incorporated into a single plasmid by fusing the sgRNA plasmid’s mCherry reporter to the Acr by a self-cleaving 2A peptide (Fig. 1b), using AcrIIC3 (demonstrated to be ineffective versus *S. pyogenes* Cas9^14^) as a null Acr. In this assay, AcrIIA4 almost completely reduced gene up- or down-regulation to zero, whereas assays performed in the presence of AcrIIC3 showed similar levels of activity as control assays (Fig. 1c–e). These results demonstrated that this 2A fusion strategy can maintain Acr function and CRISPRa and CRISPRi can be used to quantitate Acr activity.

We subsequently used this 2A fusion strategy to characterize recently discovered II-A Acr families, AcrIIA5^28^ and AcrIIA6^29^ (Supplementary Fig. 2). With this assay, we were able to determine that AcrIIA5 proteins demonstrated a modest (~80%) reduction in dCas9-induced GFP expression, consistent with a report in yeast^30^, whereas AcrIIA6 had no discernible effect.

### Acrs can inhibit CRISPRa and CRISPRi on endogenous genes

**Fig. 2:**
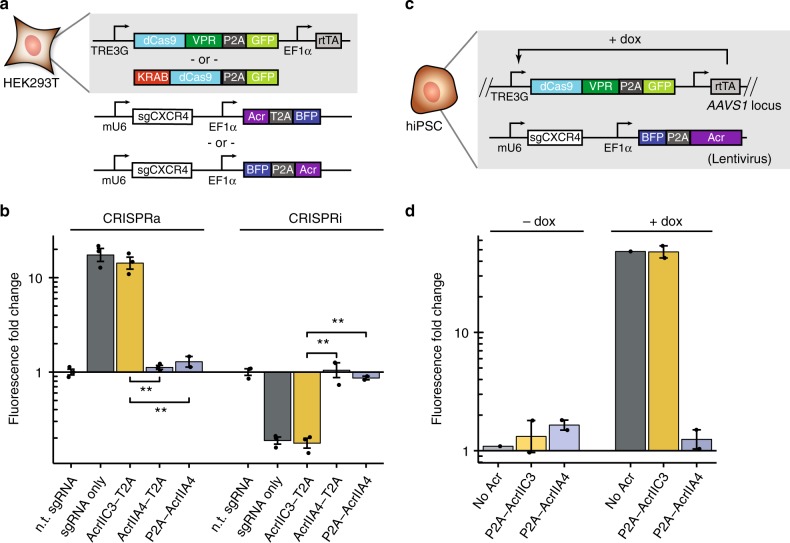
Acr regulates control of endogenous gene expression in different cell types. **a** Box diagram for endogenous gene regulation experiments in HEK293T: cells with integrated doxycycline-inducible CRISPRa or CRISPRi dCas9 effectors are transfected with plasmid encoding sgRNA and Acr fused in varying configurations to BFP via 2A peptide. **b** Change in mean expression of CXCR4 for CRISPRa and CRISPRi experiments in presence and absence of various Acr constructs for *n* = 3 experimental replicates (*n* = 2 for P2A-AcrIIA4 condition). **: *p* < 0.01. Source Data are provided as a Source Data file. **c** Box diagram of endogenous gene regulation experiments in hiPSC: cells with integrated dCas9-VPR-GFP are lentivirally transduced with sgRNA plasmids containing or lacking Acr. **d** Change in mean expression of CXCR4 in hiPSC cells without (−dox) and with (+dox) dCas9-VPR and in presence and absence of Acr for *n* = 2 experimental replicates for Acr constructs and *n* = 1 experimental replicate for “no Acr” condition. Source Data are provided as a Source Data file. Error bars indicate ± s.e.m.

We next sought to understand whether AcrIIA4 could be used in a wider variety of contexts, starting from endogenous gene regulation. We first created a set of doxycycline (dox)-inducible CRISPRa and CRISPRi PiggyBac constructs for integration into HEK293T (Fig. 2a). We used sgRNAs designed to target the expression of the endogenous C-X-C chemokine receptor type 4 (CXCR4) gene and observed strong CRISPRa (~15-fold increase) and CRISPRi (~85% decrease) activity (Supplementary Fig. 3a-b, Fig. 2b). By contrast, AcrIIA4 demonstrated almost total nullification of gene regulation activity, while AcrIIC3 showed little inhibitory effect (Supplementary Fig. 3a-b; Fig. 2b). We observed that fusing AcrIIA4 N-terminal to the 2A peptide and fluorescent protein demonstrated slightly stronger anti-CRISPR effect, possibly due to differences in the coupling efficiency of the 2A peptide^31^.

We subsequently tested the applicability of AcrIIA4 in other cell types. We used a human-induced pluripotent stem cell (hiPSC) line with our dox-inducible CRISPRa construct knocked into the AAVS1 locus (Fig. 2c). This cell line was lentivirally transduced with constructs bearing CXCR4-targeting sgRNA with and without AcrIIC3 and AcrIIA4. Consistently, we observed minimal effects on gene regulation with AcrIIC3 and strong inhibition of CRISPRa with AcrIIA4 (Supplementary Fig. 3c; Fig. 2d). This suggests AcrIIA4 works for controlling CRISPR-based gene regulation in diverse mammalian cell types as a general tool.

### Acrs inhibit CRISPR activity in yeast cells

**Fig. 3:**
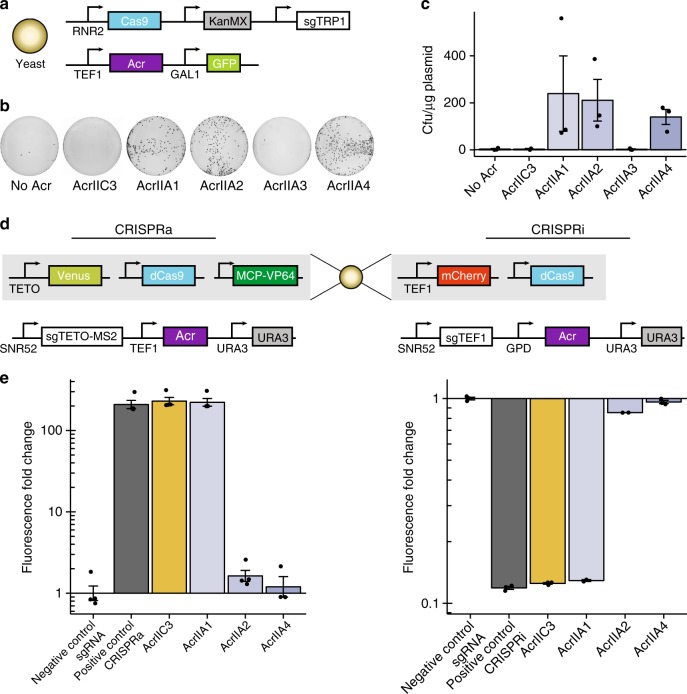
Activity of Acrs in yeast editing and gene regulation. **a** Scheme for yeast editing experiments: yeast cells are simultaneously transformed with a plasmid encoding Cas9 with sgRNA targeting essential gene TRP1 (chromosome IV) and KanMX selection marker along with a plasmid expressing Acr. **b** Representative images of colony formation on G418 plates. “No Acr” condition has a plasmid containing mCherry in the place of Acr. **c** Arithmetic means of colony formation efficiency in presence of various Acrs for *n* = 3 experimental replicates (*n* = 2 for AcrIIC3). Source Data are provided as a Source Data file. **d** Schemes for yeast gene regulation experiments: CRISPRa (left)—a yeast strain bearing stably integrated Venus reporter from a TET-inducible promoter, dCas9, and MCP-VP64 is transformed with a plasmid expressing sgRNA capable of co-localizing MCP-VP64 to the TETO promoter, as well as Acr; CRISPRi (right)—a yeast strain bearing constitutive mCherry reporter and dCas9 is transformed with plasmid expressing sgRNA targeting reporter promoter and Acr. **e** Acr-induced change in reporter expression for CRISPRa and CRISPRi experimental conditions described in **d** for *n* = 3 experimental replicates (*n* = 4 for CRISPRa conditions except AcrIIA4; *n* = 2 for CRISPRi AcrIIA1 and AcrIIA2 conditions). Source Data are provided as a Source Data file. Error bars indicate ± s.e.m.

We next investigated the efficacy of Acrs in other eukaryotic systems. *S. cerevisiae* is a common metabolic engineering platform and also provides a useful system for protein engineering. We first assessed the activity of our Acr panel using a yeast editing assay, in which yeast cells were co-transformed with a plasmid bearing Cas9, sgRNA targeting essential gene TRP1, and a KanMX selection marker alongside a plasmid bearing Acr genes (Fig. 3a). Cells could only survive on selection plates with active Acr inhibiting Cas9. Multiple type II-A Acrs were found to be effective in abrogating CRISPR activity (Fig. 3b, c), including AcrIIA1, which showed minimal effect in our mammalian CRISPRa and CRISPRi assays or previously described CRISPRi and editing assays^15^.

To further understand the function of Acrs in yeast, we also tested their activities on CRISPRa and CRISPRi using a pair of reporter strains and transforming a plasmid bearing sgRNA and Acr (Fig. 3d). Consistently, we found that plasmids containing AcrIIA3 were toxic, even when transformed without dCas9 (Supplementary Fig. 4a). AcrIIA3 was previously found to be toxic in bacteria^15^, indicating that a common mechanism may underlie its biological effects. Other Acrs demonstrated a consistent effect on CRISPRa and CRISPRi (Supplementary Fig. 4b; Fig. 3e). Interestingly, all tested II-A Acrs were effective, except for AcrIIA1. These assays in concert suggest the following results: AcrIIA1 is a strong inhibitor of Cas9 editing in yeast but not dCas9-based gene regulation, implying a possible mechanism of inhibition at the editing level (perhaps akin to a previously reported mechanism for a II-C Acr^18^); and AcrIIA2 demonstrates stronger apparent activity for gene editing and gene regulation in yeast than in mammalian cells (consistent with other reports^30,32^).

### Acrs negate activity of CRISPR-based synthetic devices

**Fig. 4:**
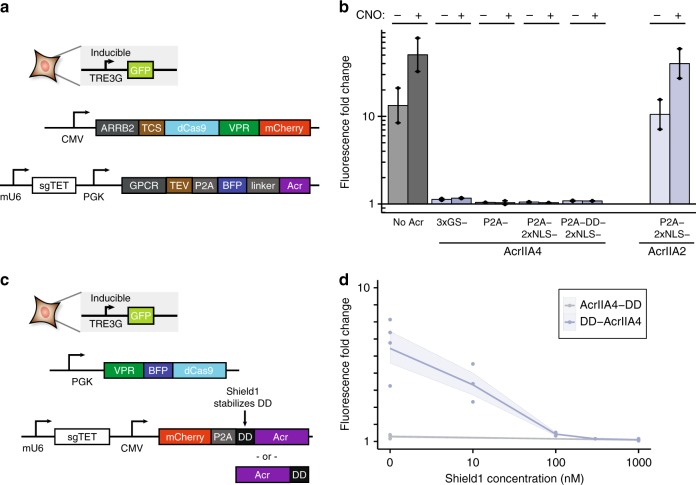
Acr activity in inducible CRISPRa contexts. **a** Box diagram of GPCR activation experiment: a reporter cell line is transiently transfected with plasmids encoding sgRNA, synthetic GPCR with TEV protease and Acr, and β-arrestin-2 fused to dCas9-VPR via a TEV cleavage site (TCS). **b** Change in reporter fluorescence with various Acr constructs in absence and presence of GPCR-activating ligand clozapine-N-oxide (CNO) for *n* = 2 experimental replicates. Source Data are provided as a Source Data file. **c** Diagram of Shield1-inducible control experiment: a reporter cell line is transiently transfected with CRISPRa VPR-dCas9 plasmid along with sgRNA plasmid containing fusions of AcrIIA4 to Shield1-stabilized destabilization domain (DD). **d** Inducible control of gene activation from DD-AcrIIA4 fusions as a function of stabilization reagent Shield1 for *n* = 3 experimental replicates (*n* = 4 for DD-AcrIIA4 0 nM and 1000 nM conditions, *n* = 2 for 100 nM condition, and *n* = 1 for 300 nM condition). Source Data are provided as a Source Data file. Shaded region indicates ± s.e.m.

We subsequently examined the use of Acrs in inducible gene regulation systems. We recently reported a G-protein-coupled receptor (GPCR)-activated dCas9 gene regulation system (ChaCha system)^8^, which combines a GPCR fused to tobacco etch virus protease (TEV) and V_2_ vasopressin tail and a dCas9-VPR fused to β-arrestin via a TEV cleavage site (TCS). GPCR activation by cognate ligand binding induces recruitment of β-arrestin, which allows for release of dCas9-VPR for nuclear localization and gene activation. We incorporated AcrIIA4 into the ChaCha system via a variety of linkers—3xGlySer, P2A, nuclear localization signal (NLS) and destabilization domain (DD)—downstream of the GPCR construct (Fig. 4a). While control ChaCha assays demonstrated ligand-inducible gene activation, all of our AcrIIA4 fusions completely inhibited the synthetic device in both on and off states (Fig. 4b).

To further understand the nature of this effect, we tested the same set of constructs with a free dCas9-VPR control (Supplementary Fig. 5a) and again obtained significant reductions in activation (Supplementary Fig. 5b). By contrast to our Acr ChaCha and previous Acr CRISPRa assays, there remained residual (3- to 8-fold) activation. By comparing the performance of the identical (after cleavage) P2A-AcrIIA4 construct in this and our previous Acr CRISPRa assays, these results can most likely be at least partially explained by the difference in stoichiometric ratios: the CMV (strong) and PGK (weak) promoters are switched (and the GPCR-TEV-P2A-BFP construct is much larger than the mCherry fused to AcrIIA4 in Fig. 1). This idea that relative stoichiometry may play a role in overall activity was corroborated by an experiment wherein dCas9-VPR and Acr are produced from the same plasmid in roughly a 1:1 ratio (Supplementary Fig. 5c), resulting in similar amounts of significant, but not total, downregulation of gene expression (Supplementary Fig. 5d).

Based on these combined results, the performance of various Acr fusions can be assessed: fusing AcrIIA4 to NLSx2 seems to diminish its performance, even relative to a BFP fusion (GSx3 linker), and, in accordance with expectations, fusing DD reduces performance further. Similarly, the stronger performance of these construct variants on the ChaCha assay relative to the free dCas9-VPR assay seems to imply that active free dCas9-VPR molecules in the ChaCha assay (after protease cleavage) are produced in lower amounts relative to constitutive expression of the free dCas9-VPR. We also tested a P2A-NLSx2-AcrIIA2 construct, which showed no inhibition of CRISPRa even in the ChaCha assays, implying that this construct is almost completely inactive. The results suggest that switching between experimental or construct configurations might allow for deeper quantitative understanding of both CRISPR-based devices and anti-CRISPR activity.

### Inducible Acrs allows dosable control of CRISPR activity

We further engineered Acrs for inducible control of gene expression. To do this, we fused an engineered inducible destabilization domain (DD)^33^ to AcrIIA4 (Fig. 4c). Addition of cognate ligand Shield1 stabilizes DD and the fused AcrIIA4, leading to stronger inhibition of dCas9 activity. We tested the DD-AcrIIA4 constructs using our reporter CRISPRa assay and observed Shield1-dependent switch behavior (Fig. 4d), while normal CRISPRa or Acr lacking DD showed no response (Supplementary Fig. 6a,c). Fusing DD to the N-terminus of AcrIIA4 showed Shield-1-inducible gene expression, while fusing DD to the C-terminus was ineffective, possibly due to the reduced amount of induced degradation provided by DD at the C-terminus^34^. We also observed no change in activation with a DD-VPR-dCas9 construct (Supplementary Fig. 6b,c). A DD-Cas9 was previously shown to have inducible editing activity;^35^ the difference possibly lies in the increased size of the VPR-dCas9 construct and the delivery method (transient transfection versus viral transduction). Notably, another DD variant was found to be ineffective in modulating gene expression when fused to dCas9 in another study^12^, further corroborating that DD provides incomplete control over dCas9-based effector function. These results demonstrate the utility of AcrIIA4 as an easily incorporable and modular tool for engineering inducible dCas9 activity, requiring no re-engineering of the dCas9 construct (and demonstrating superior performance in at least certain contexts).

### Acrs offer a means to genomically write-protect cells

With the broad use of CRISPR for multiple purposes in diverse organisms, including gene editing to correct diseases, generation of genetically modified organisms (GMOs), and field testing of gene drives to eliminate species, it remains a major need to devise new countermeasures that provide prophylactic options to limit genome editing in organisms and protect genome integrity in populations of organisms. Therefore, we tested whether human cells pre-engineered with Acr molecules become resistant to gene editing, which results in a genome with “write protection” against specific Cas9s.

**Fig. 5:**
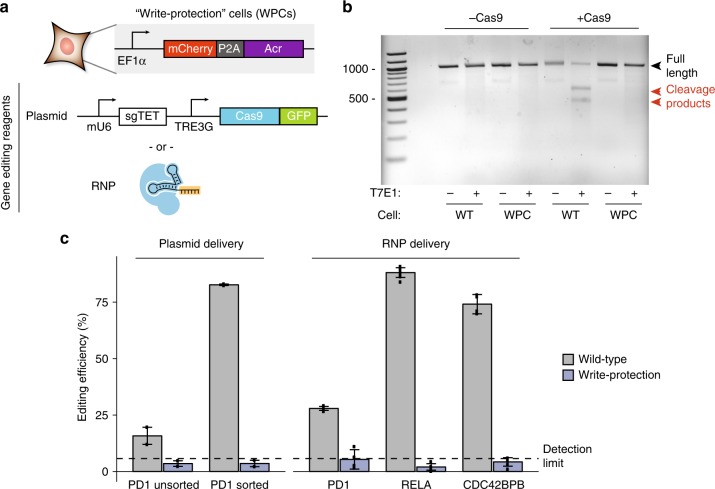
Cells with integrated AcrIIA4 become “write-protected” against future editing. **a** HEK293T cells were lentivirally transduced with a cassette constitutively expressing AcrIIA4. A clonal line was isolated and compared to untransduced wild-type cells. Cas9 + sgRNA was delivered either by a plasmid expressing both components or purified ribonucleoprotein complex (RNP). **b** Results from a T7E1 assay comparing editing efficiency between wild-type (WT) and AcrIIA4 (WPC) line targeting the *PD1* locus run on an agarose gel. 100 bp standard is marked on left lane with positions of 500 and 1000 bp bands noted. Predicted lengths for uncut (996 bp) and cleaved products (437 and 559 bp) are annotated. Source Data are provided as a Source Data file. **c** Editing efficiency as quantified by TIDE analysis, with plasmid delivery, unsorted (plasmid) and sorted (sorted), as well as RNP delivery at various genomic loci. Error bars indicate s.e.m. of *n* = 2 experimental replicates for plasmid delivery and an estimate of technical variance (s.d.) of a single experimental replicate for RNP delivery. The dotted line is an estimate of detection sensitivity computed from sequencing traces of unedited cells (mean + 2× s.d.). Source Data are provided as a Source Data file

We first tested the efficacy of AcrIIA4 in a HEK293T reporter system for gene editing and noted that co-transfection of AcrIIA4 plasmid resulted in strong, but not total inhibition of gene editing (Supplementary Fig. 7). We then stably integrated a lentiviral construct encoding AcrIIA4 into the genome of HEK293T cells (Fig. 5a), generating write-protected cells (WPCs). We tested gene editing in WPCs compared to wild-type HEK293T cells by delivering Cas9 and a sgRNA targeting various genomic loci. Using a plasmid delivery method followed by T7E1 assay, we observed no editing in WPC cells pre-engineered with AcrIIA4, while wild-type HEK293T cells exhibit strong gene editing (Fig. 5b). We further compared various delivery methods using plasmids, with or without subsequent sorting, and ribonucleoprotein complexes (RNPs), and in all cases gene editing in AcrIIA4-engineered WPC cells was negligible with a similar level below the detection limit of Tracking of Indels by Decomposition (TIDE) analysis^36^ (Fig. 5c). Our data prove the feasibility of this approach of generating “write protection” in cells to counteract further gene editing, thus laying a foundation to prevent accidental or intentional gene editing in desired cell types.

### Acr-based genetic circuits provide pulsatile gene expression

**Fig. 6:**
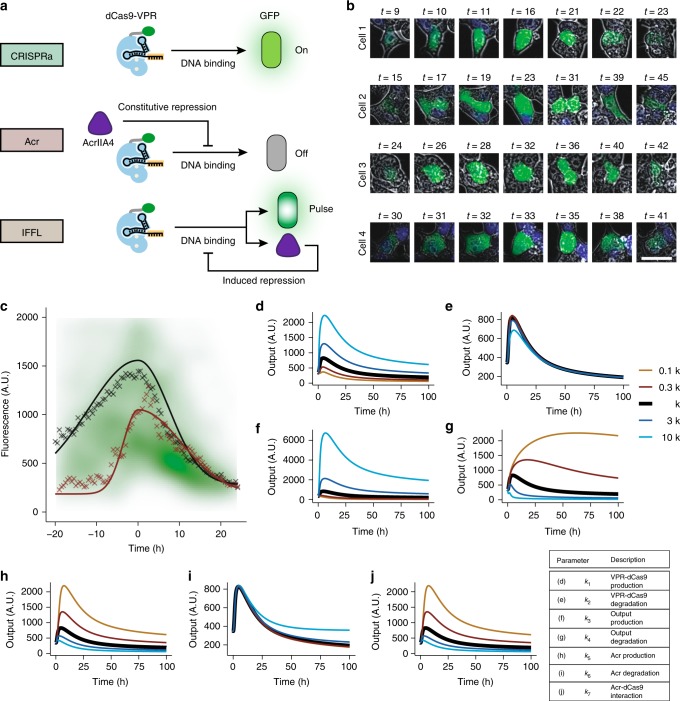
Pre-programmed Acr-based genetic circuits. **a** Genetic circuits analyzed via live-cell microscopy. CRISPRa: dCas9-VPR drives inducible GFP reporter expression; Acr: constitutive expression of AcrIIA4 prevents dCas9-VPR-based CRISPRa activity; IFFL: dCas9-VPR simultaneously drives inducible GFP and AcrIIA4 expression, resulting in a pulse of activity. **b** Selected snapshots of cell-tracking traces of the IFFL circuit. Each row corresponds to a single trace, with time post-transfection annotated above each frame. Scale bar in lower right corresponds to 30 µm. **c**–**j** Experimental and computational exploration of IFFL circuit activity. Circuit activity (*y*-axis) corresponds to GFP production in **c** and computed response for an arbitrary circuit output for **d**–**j**. **c** Aligned activity of IFFL condition. Shown are the time-dependent median expression of GFP of cell tracking traces for two separate experiments (black and red points). Overlaid are median fits (solid lines) and the combined density plots of both experiments (green), encompassing *n* = 187 cell traces. *t* = 0 corresponds to aligned maximum of pulse for each trace. Source Data are provided as a Source Data file. **d**–**j** Parameter sensitivity analysis from computational modeling. Plotted are predictions of behavior of the IFFL circuit when changing **d** dCas9-VPR production rate; **e** dCas9-VPR degradation rate; **f** dCas9-VPR-dependent output production rate; **g** output degradation rate; **h** dCas9-VPR-dependent Acr production rate; **i** Acr degradation rate; **j** dCas9-Acr interaction rate. The thick black line for each plot corresponds to computed activity for the value of that parameter derived from fits to experimental data, while other lines correspond to alterations to that parameter plus or minus one order of magnitude. *t* = 0 corresponds to activation of circuit response

Another potential advantage of Acr-based control of gene regulation is that it lends itself as a method to implement pre-programmed genetic circuitry. To test this idea, we generated 3 simple gene regulation circuits (Fig. 6a): CRISPRa (VPR-dCas9-driven GFP expression), Acr (VPR-dCas9 activity inhibited by constitutive Acr expression), and an incoherent feedforward loop (IFFL) circuit (VPR-dCas9 driving both GFP and Acr expression, resulting in delayed abrogation of CRISPRa activity). To observe the effects of these circuits in a time-dependent manner, we stably integrated a plasmid containing sgRNA and appropriate Acr construct and triggered the circuit via transient transfection of VPR-dCas9 plasmid (Supplementary Fig. 8a). The resulting changes in expression of GFP were tracked by live-cell time-lapse microscopy. Though individual cells exhibited heterogeneous responses, clear qualitative differences in phenotypes among the different circuits were evident (Supplementary Movies 1–3).

To more quantitatively understand the behavior of the circuits, we implemented an unbiased automated cell tracking image analysis pipeline, allowing us to follow the GFP trajectory of a cell (or a cluster of cells) over time (Fig. 6b; Supplementary Movie 4). By tracking the GFP fluorescence of these cells, we observed clear CRISPRa and anti-CRISPRa phenotypes in the combined population for the CRISPRa and Acr conditions, while the IFFL condition demonstrated intermediate GFP expression (Supplementary Fig. 8b). In contrast to the CRISPRa and Acr circuits, expression of mCherry remained low throughout activation of the IFFL circuit (Supplementary Fig. 8c), suggesting that a relatively small amount of AcrIIA4 is sufficient to effect reversal of CRISPRa activity in the IFFL case.

We hypothesized that the GFP signal in the bulk population from the IFFL circuit was diluted by variation in the onset time of the circuit, wherein different cells activate at different times (Fig. 6b; Supplementary Movies 3–4), most likely due to biological variation and stochastic differences in delivery time of the circuit-activating VPR-dCas9 plasmid. To account for this variation, we fit each cellular trajectory to an asymmetric gaussian function, allowing for the alignment of these traces in time relative to the peak point of response. The aligned traces demonstrated a pulse-like phenotype of GFP expression for the IFFL condition (Fig. 6c), whereas a similar alignment procedure revealed sigmoidal behavior with high or low amounts of GFP expression for the CRISPRa and Acr conditions, respectively (Supplementary Fig. 8d).

Based on fits to the IFFL condition, we estimated descriptions of average circuit response, including pulse width (half-rise time ~6 h, half-decay time ~10 h) and amplitude (peak expression ~5-fold relative to basal level). We also tested the IFFL condition without stably integrating the sgRNA plasmid and observed similar pulse behavior in a percentage of cells (Supplementary Fig. 8e), indicating the possibility of achieving programmed circuit behavior solely via transient delivery of DNA. The ability to implement “pulse generator” circuits using Acrs highlights their utility in synthetic circuit engineering, which could provide a powerful method to control genes in a highly programmable and dynamic manner.

We built a simple computational model in order to further understand the basis of the IFFL circuit behavior (Supplementary Methods). We parameterized the model via sequential fits to experimental data and were able to recapitulate the qualitative behavior of all three implemented circuits (Supplementary Fig. 9). We subsequently generated theoretical predictions of the effects of perturbing various parameters on the performance of the circuit (Fig. 6d–j). Notably, parameters such as VPR-dCas9 and Acr production rate, as well as Acr strength of interaction, were predicted as major factors that control amplitude of the generated pulse. These results provide semi-quantitative predictions of how circuit behavior may be rationally altered and lay the groundwork for further implementation of genetic circuits with tunable dynamics and responses.

## Discussion

In this work, we evaluated and characterized a panel of natural phage-derived anti-CRISPR proteins using both CRISPRi and CRISPRa in mammalian cells. Our results demonstrated that AcrIIA4 is a potent regulator of (d)Cas9 activity in a wide variety of contexts (reporter or endogenous genes) and cell types (HEK293T, hiPSC, and yeast). The small size of AcrIIA4 allows it to be incorporated in a range of contexts easily, and it remains highly efficient in inhibiting CRISPR activity when fused to other gene products via many linkers including 2A peptides at either the N- or C-terminus, or larger domains such as DD or fluorescent proteins. We also demonstrated that AcrIIA4 is effective in ablating CRISPR-based complex synthetic devices. Fusing a destabilization domain to AcrIIA4 enabled tunable and inducible control of CRISPRa activity. This, combined with other reports^14,15,21^, contributes to a picture on the use of Acrs as an additional layer of control over (d)Cas9 activity in eukaryotic cells. Thus, the work presented here expands the existing CRISPRa / i tool set by characterizing a useful and tunable inhibitor molecule, which is useful for probing biology in a wider range of contexts.

The advent of CRISPR technologies has drastically reduced the barrier of genome editing and control over gene expression. While undoubtedly a boon to science, this ease of use has also raised concomitant fears of the use of gene editing for malignant or otherwise unethical or illegal purposes. Our results are a proof-of-principle demonstration of the practicality of integrating Acrs in the genome to generate cells that are immune to unlicensed editing applications. We also observed that AcrIIA4 could operate when present at low-copy number (genomically integrated versus transiently transfected), and there was no apparent cytotoxic effect nor loss of expression of Acr over months of culturing in HEK293T and hiPSC lines. AcrIIA4 inhibits *Spy* (d)Cas9, by far the most commonly used CRISPR system, but potentially may be bypassed with another Cas9 ortholog. However, due to the small size of most Acrs, we believe it would be possible to create a cassette encoding multiple Acrs targeting commonly used orthologs, especially as the wealth of Acrs becomes uncovered^37–39^ or to use promiscuous anti-CRISPRs^18^ as broad-spectrum inhibitors.

Based on our results, we anticipate that an integration approach should also work for defending against Cas9-based editing in *S. cerevisiae*; this may be of use to preserve the integrity of lines used in the production of sensitive materials including toxic products and controlled substances^40^. Additionally, we propose that genomically write-protected organisms may be used as a safety valve for counteracting CRISPR-based gene drives. Cas9-driven gene drives have already been implemented in organisms such as mosquitos^41,42^, but one major concern is the potential for unforeseen ecological impacts resulting from population collapse. We believe that the introduction of a population with an integrated Acr should inhibit and possibly allow for the reversal of the spread of the gene drive; indeed, a proof-of-principle study demonstrated the use of Acrs for the inhibition of a gene drive in yeast^32^.

The use of CRISPRa and CRISPRi for assessing Acr activity relative to editing assays affords a few advantages: the assays are quick and can provide quantitative information on Acr activity (rather than a discrete readout of edited / not-edited) on the single-cell level, and thus are ideal for assessing Acr activity in eukaryotic cells. Further, the combination of CRISPRa and CRISPRi assays (in addition to editing assays) allows for distinguishing between specific anti-CRISPR activity and potential cytotoxic effects and revealed that other Acrs demonstrate varying activity depending on the organismal and CRISPR activity (knockout versus gene regulation) context. These results suggest differences in underlying mechanisms in the various Acr families and open up new lines of inquiry in exploring Acr function. Our results suggest that we can begin to move from a regime of classifying Class-II-targeting Acrs in a binary manner as effective or non-effective into one where anti-CRISPR activity can be more finely determined. We believe that these assays, coupled with the feasibility of using such organisms as *E. coli* and *S. cerevisiae*, allow for rapidly screening of discovered or designed Acrs for enhanced or tuned activity, activity targeting Cas9 variants and orthologs, and promiscuity of Acr activity; based on the mechanism of Cas9, novel Acrs that can modulate dCas9 activity should also prove sufficient for inhibiting Cas9-driven editing.

The use of Acrs in regulating CRISPR activity should allow for the generation of more advanced dynamic control over gene regulation. Our results demonstrate that incorporating inducible control over AcrIIA4 can be superior to using the same technology directly on Cas9. It is possible that the temporal dynamics of Acrs under such methods of control would be sharper compared to control over Cas9, because Acr is smaller and can be likely produced and degraded more quickly. The level at which Acrs operate is distinct from other methods of inducible control^7,8^, allowing for multilayered logical control over dCas9-based gene regulation. A recent report also demonstrated the utility of incorporating control on the Acr by realizing light-dependent activity of Acr^20^, further cementing this notion of Acr as an easily adaptable mode of control over (d)Cas9 function. Furthermore, as inducible control over Acrs and Cas9 function have opposite relationships, it is likely that implementing control over both Acrs and Cas9 will allow for inversion of the response of Cas9 activity relative to the control signal. We suggest that Acrs will serve as a useful part of developing new methodologies governing CRISPR activity.

We demonstrate in this report a proof-of-concept pulse generator circuit implemented via Acr-dCas9 interaction. Although a similar sort of circuit was previously reported in bacteria^43^, to our knowledge, a synthetic pulse response circuit has not been reported in mammalian cells. Utility of these circuits may be enhanced by adding additional nodes of control (such as dCas9 orthologs/Acrs or other gene regulation tools) and implementing control over endogenous genes, as well as computational modeling to analyze and optimize circuit performance. Our work opens the door to use dCas9 and Acrs to build dynamic pre-programmed gene regulation circuits and to understand the behavior of these circuits in a more quantitative manner. With further investigation, Acrs are poised to enter the realm of quantitative synthetic biology, potentially integrating multiple inputs and dCas9 effectors into genetically encoded gene regulation programs.

## Methods

### Materials

Plasmids and cell lines were generated using standard molecular cloning techniques. See Supplementary Tables 1–4 for details on constructs used.

### Cell culture

HEK293T cells (Clontech) were cultured in DMEM + GlutaMAX (Thermo Fisher) supplemented with 10% Tet-FBS (Clontech). Human iPSCs were cultured in mTeSR (STEMCELL Technologies). Cells were maintained and passaged using standard cell culture techniques and were maintained at 37 °C and 5 % CO_2_. Cells were not regularly monitored for mycoplasma contamination.

Transient transfections were performed using TransIT-LT1 transfection reagent (Mirus). Lentivirus for generating reporter cell lines and sgRNA transduction was packaged using wild-type HEK293T (Clontech). Lentiviral transduction was performed at roughly 0.25 multiplicity-of-infection. Transduced HEK293T cell lines were sorted in bulk (except for the write-protected cell line, which was clonally sorted). The cell line containing the IFFL was sorted for negative mCherry expression, then transfected with a plasmid bearing tetracycline-controlled transactivator (tTA) and sorted for positive expression 2 days post transfection. Doxycycline-inducible KRAB-dCas9 and dCas9-VPR HEK293T stable lines were generated using the PiggyBac transposon system. dCas9-VPR and rtTA were knocked into the *AAVS1* locus in previously generated hiPSCs^44^ using techniques as previously described^45^; briefly, plasmids bearing TALENs targeting the *AAVS1* locus were co-transfected along with plasmid containing the VPR-dCas9 + rtTA construct using the Amaxa Nucleofection system and a clonal population isolated.

Activation experiments were assessed for activity 2 days post transfection; repression experiments were assessed at 5 days. Doxycycline-inducible constructs were maintained in medium supplemented with 1 µg/mL doxycycline starting from the transfection date (except for −dox conditions). For GPCR experiments, cells were exposed to 20 µM ligand clozapine-N-oxide for 1–2 days before assessing activity. For DD-Shield1 experiments, medium of cells was supplemented with appropriate amount of Shield1 ligand immediately following transfection.

### Gene regulation flow cytometry and analysis

For CXCR4 expression analysis, cells were targeted with APC-labeled CXCR4 antibody (BioLegend #306510) before flow cytometry assays. Analysis of flow cytometry data, including compensation, was performed using FlowJo. Cells were gated for viability and single cells, as well as positive fluorescence markers. Fluorescence values were normalized within experimental runs to an untransfected control. For plotting, values were normalized to the non-targeting sgRNA negative control condition, except for hiPSC data, which were normalized to the +dox, no sgRNA condition. Unless otherwise stated, average values provided are geometric means and error bars are ±s.e.m. Significance tests and *p*-values were calculated using two-sided Welch’s *t*-tests.

### Mammalian editing experiments

For gain-of-function editing experiments, a HEK293T reporter cell line with out-of-frame split-GFP construct was transfected with Cas9 and sgRNA plasmid with and without plasmid containing AcrIIA4. Three days after transfection, cells were analyzed via flow cytometry without gating for presence of plasmid.

For write-protection editing experiments, HEK293T cells were transiently transfected with plasmid bearing Cas9-eGFP and sgRNA, as above, or Cas9 protein (IDT or Synthego) with synthetic modified sgRNA (Synthego) via electroporation (Invitrogen Neon or Amaxa Nucleofection). Plasmid transfections were sorted 1–2 days post-transfection for presence of GFP. Genomic DNA was isolated via spin-column chromatography (Qiagen), amplified at target locus via PCR (Kapa Biosystems), and assessed via T7E1 endonuclease activity (NEB) or TIDE sequencing analysis^36^. Sequencing traces (Quintara Biosciences) were compared to untransfected conditions to determine editing efficiency; sequencing traces of untransfected conditions were compared to each other to determine baseline noise of the TIDE assay.

### Microscopy data and analysis

Cells for microscopy were cultured in FluoroBrite DMEM medium supplemented with GlutaMAX (Thermo Fisher) and 10 % Tet-FBS. Cells were transfected as above, and immediately placed in a microscopy chamber pre-equilibrated and maintained at 37 °C and 5% CO_2_. Imaging was performed with an integrated Leica microscopy system. Selected fields of view were automatically imaged for mCherry, GFP, and BFP fluorescence and combined into time series post-acquisition.

Movies were background-subtracted (ImageJ), and cell-centered traces were generated by an automated algorithm for computationally tracking spots in the GFP channel and joining identified spots into combined tracks (TrackMate). Spots were filtered by threshold and signal-to-noise ratio, and traces comprising fewer than 12 spots were automatically discarded. Cell-centered sub-movies were generated on remaining traces and analyzed via a custom Python script. To calculate fluorescence for each trace, a 31 pixel (~10 µm) square region was isolated, and the arithmetic mean of the top 50 % highest intensity pixels was calculated for each frame of the sub-movie. To perform the fit, these traces were fit to an asymmetric gaussian function using a trace-specific *t*_0_ (center of gaussian) and amplitude and global minimum value and rate constants governing decay to the left and right of the center of the curve. To account for spurious detection and low-signal traces, we applied an iterative filtering process, discarding any traces that had amplitude less than the minimum value (i.e., lower than 2-fold change) and then re-performing the fit until convergence.

### Yeast experiments

For editing experiments, competent yeast stocks (CEN.PK2–1D strain) were generated and transformed with standard protocols (Frozen-EZ Yeast Transformation II Kit, Zymo Research) and were grown at 30 °C in yeast peptone dextrose (YPD) liquid medium supplemented with 80 mg/L adenine hemisulfate and YPD-agar plates with 1 g/L monosodium glutamate and 400 mg/L G418 sulfate. Transformants were recovered for 2 h in YPD before plating with G418 selection. Colony formation was assessed 48–60 h after plating.

For toxicity experiments, CEN.PK2-1D was transformed with Acr plasmids alone, as above, plating on synthetic complete agar plates lacking uracil. For CRISPRa and CRISPRi experiments, reporter strains yJZC10 and yJZC14, respectively, were transformed with plasmids, as toxicity experiments. Overnight cultures were back-diluted 1:4 in synthetic complete medium lacking uracil and assessed 4–6 h later via flow cytometry. Control conditions (strains lacking plasmid) were transformed and grown in media containing uracil.

### Reporting summary

Further information on experimental design is available in the Nature Research Reporting Summary linked to this article.

## Supplementary information

Supplementary Information

Description of Additional Supplementary Files

Supplementary Movie 1

Supplementary Movie 2

Supplementary Movie 3

Supplementary Movie 4

Reporting Summary

Source Data

## Data Availability

The data and code that support the findings of this study are available upon request to the corresponding author (L.S.Q.).
