# Supplementary material for: Anti-CRISPR-mediated control of gene editing and synthetic circuits in eukaryotic cells
Source: Nat Commun. 2019 Jan 14;10:194. doi: 10.1038/s41467-018-08158-x (PMC6331597; doi:10.1038/s41467-018-08158-x)
Supplement: Supplementary file 1 — Supplementary Information [file 41467_2018_8158_MOESM1_ESM.pdf]

Supplementary Information

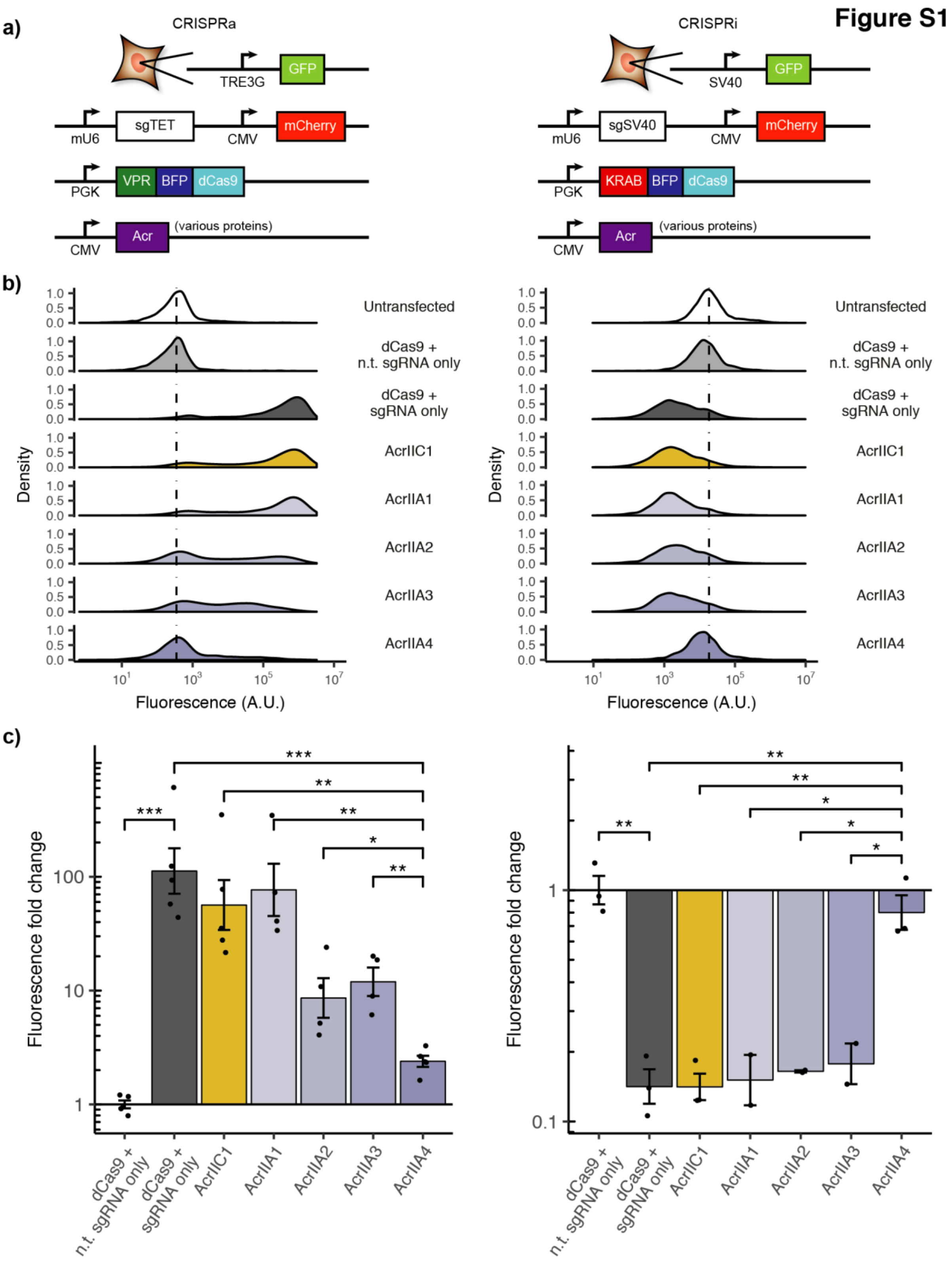

Supplementary Figure 1: CRISPRa and CRISPRi reveal differences in Acr activity

a) Experimental schemes for assessing Acr variant activity for CRISPRa (left) and CRISPRi (right): reporter cell lines are transfected with plasmids encoding sgRNA, dCas9-effector, and Acr. **b**) Representative raw flow cytometry traces of reporter fluorescence in presence of various Acrs. Dotted line indicates median fluorescence of untransfected condition. **c**) Summary of effect of panel of Acrs on CRISPRa (left) and CRISPRi (right). (*: $p < 0.05$; **: $p < 0.01$; ***: $p < 0.001$) for n=5 (CRISPRa; n=4 for AcrIIA1, AcrIIA2, and AcrIIA3) and n=3 (CRISPRi; n=2 for AcrIIA1, AcIIA2, AcrIIA3) experimental replicates. Source data are provided as a Source Data file. Error bars indicate ± s.e.m.

Figure S2

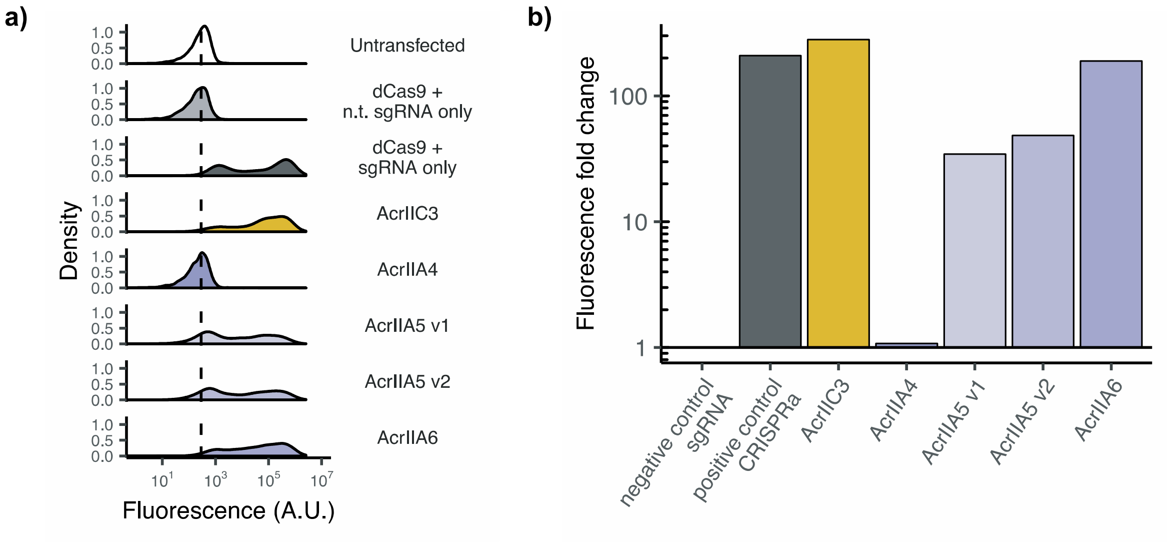

Supplementary Figure 2: comparison of activities of AcrIIA5 and AcrIIA6

Results from a dual-plasmid CRISPRa experiment comparing additional Acr families. Plasmid bearing sgRNA + Acr variant was co-transfected with dCas9-VPR plasmid. **a)** Raw flow cytometry traces of GFP fluorescence from a single experimental replicate. Dotted line indicates median fluorescence of untransfected condition. **b)** The mean fluorescence for each condition normalized to the negative control sgRNA condition. Source data are provided as a Source Data file.

Figure S3

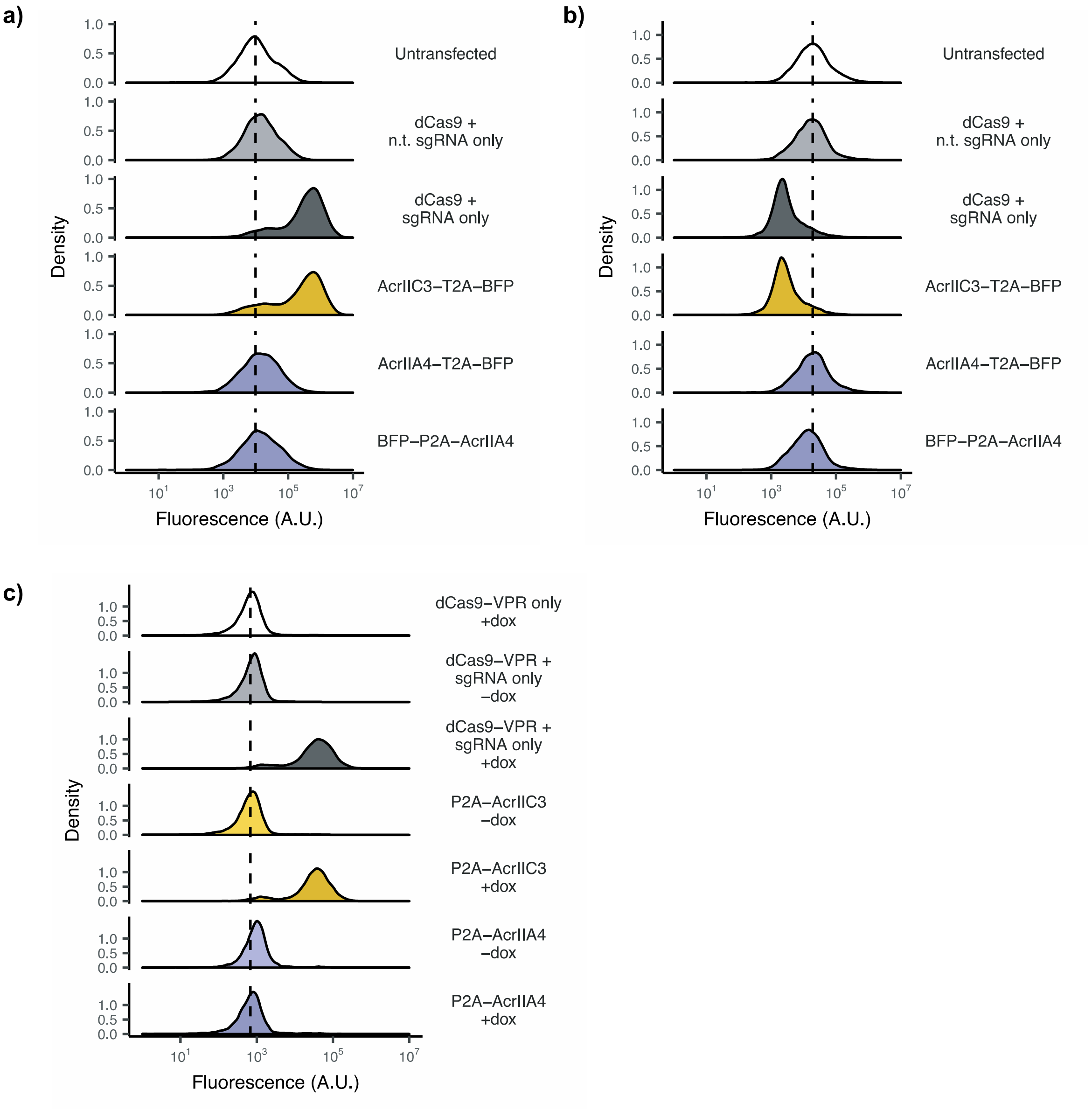

Supplementary Figure 3: AcrIIA4 regulates CRISPRa and CRISPRi on endogenous CXCR4 expression

a-b) Representative raw fluorescence flow cytometry traces of CXCR4 immunostaining for HEK293T CRISPRa (**a**) and HEK293T CRISPRi (**b**). Dotted line indicates median fluorescence of untransfected condition. **c)** CXCR4 CRISPRa in hiPSC. Traces compare conditions in absence (-dox) and presence (+dox) of dCas9-VPR effector. Dotted line indicates median of dCas9-VPR-only cell line (+dox).

Figure S4

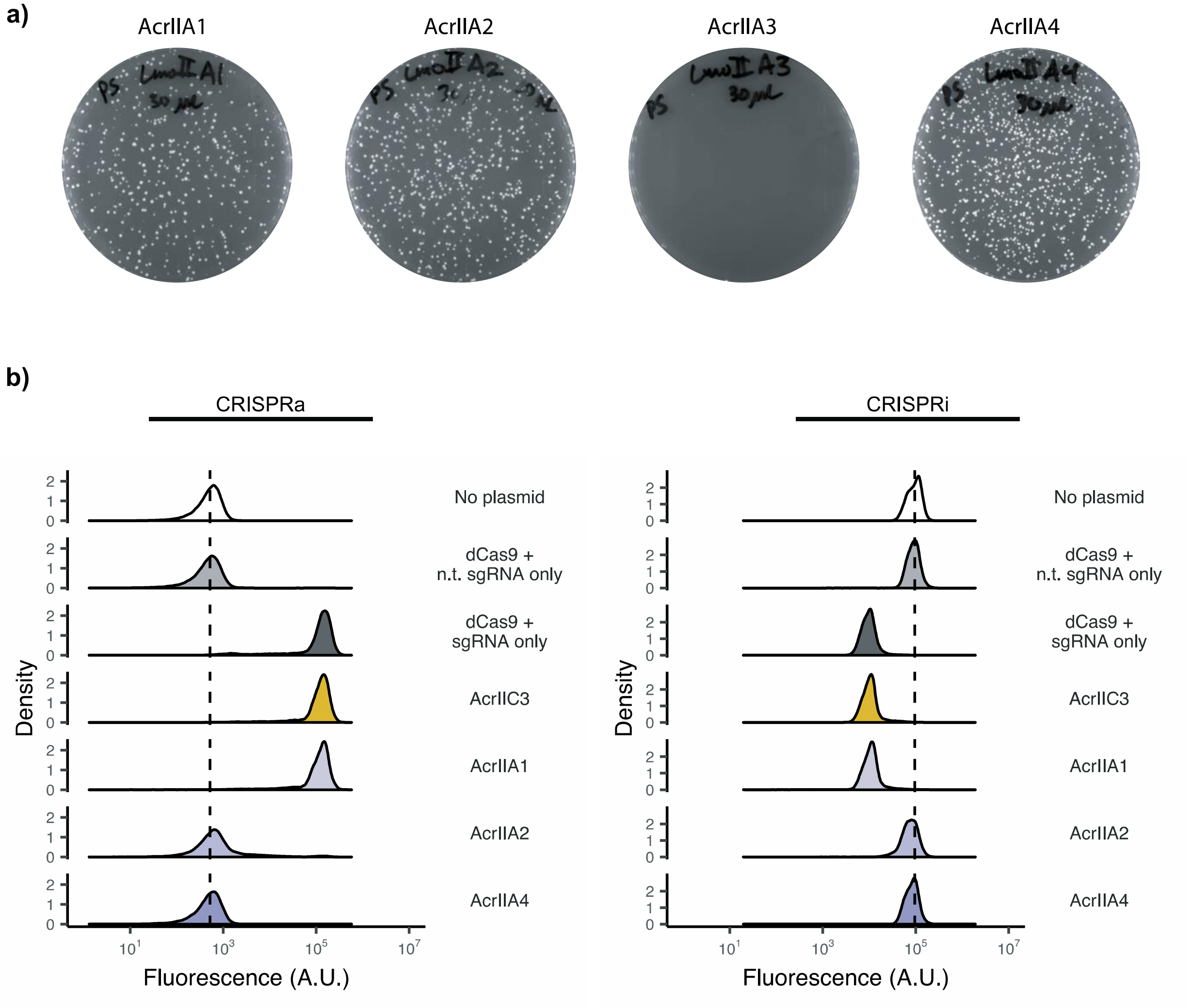

Supplementary Figure 4: Acrs demonstrate varying activity depending on experimental context

a) Toxicity of Acrs in yeast. Yeast were transformed with plasmids bearing Acr variants and grown under uracil auxotrophic conditions. AcrIIA3 demonstrates a clear toxic phenotype compared to other Acrs tested. **b)** Representative raw flow cytometry density traces of reporter expression for CRISPRa and CRISPRi experiments in yeast.

Figure S5

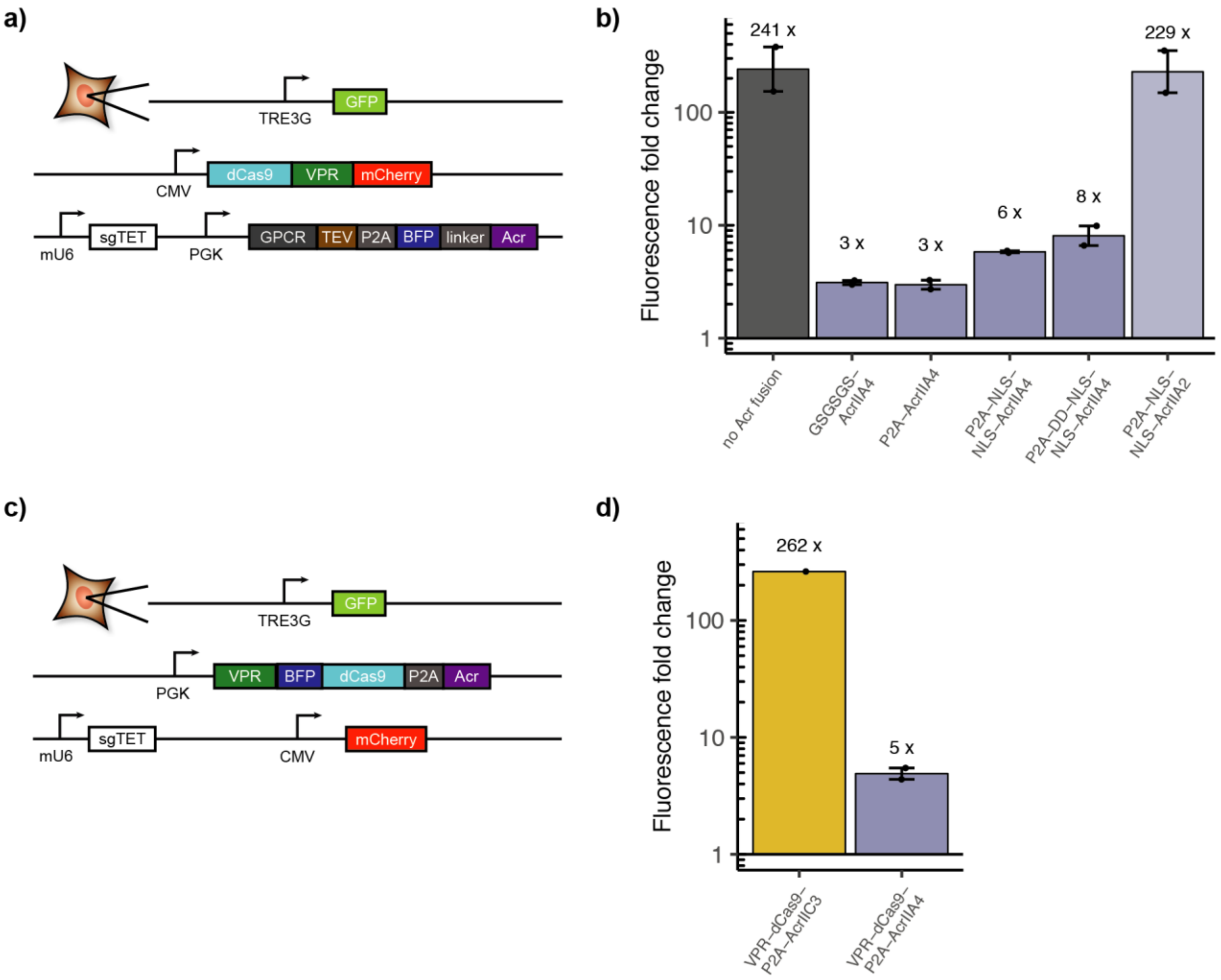

Supplementary Figure 5: Acr activity depends on stoichiometric ratio and fusion context

a) Schematic for free dCas9 controls: a reporter cell line is transiently transfected with a plasmid encoding sgRNA, synthetic GPCR with TEV protease, and Acr simultaneously with one encoding dCas9-VPR. **b**) Summary of activity from n=2 experimental replicates of various Acr fusions under conditions from (**a**). Fold changes in GFP expression are labeled for each condition. Source data are provided as a Source Data file. **c**) Acr:VPR-dCas9 expressed at roughly 1:1 ratio: reporter cells are transiently transfected with sgRNA plasmid and a construct fusing Acr to VPR-dCas9 via a P2A self-cleaving linker. **d**) Comparison of Acr activity of constructs from the experiment described in (**c**). Fold changes in GFP expression are labeled for each condition. n=1 experimental replicate for AcrIIC3 and n=2 for AcrIIA4 condition. Source data are provided as a Source Data file. Error bars indicate ± s.e.m.

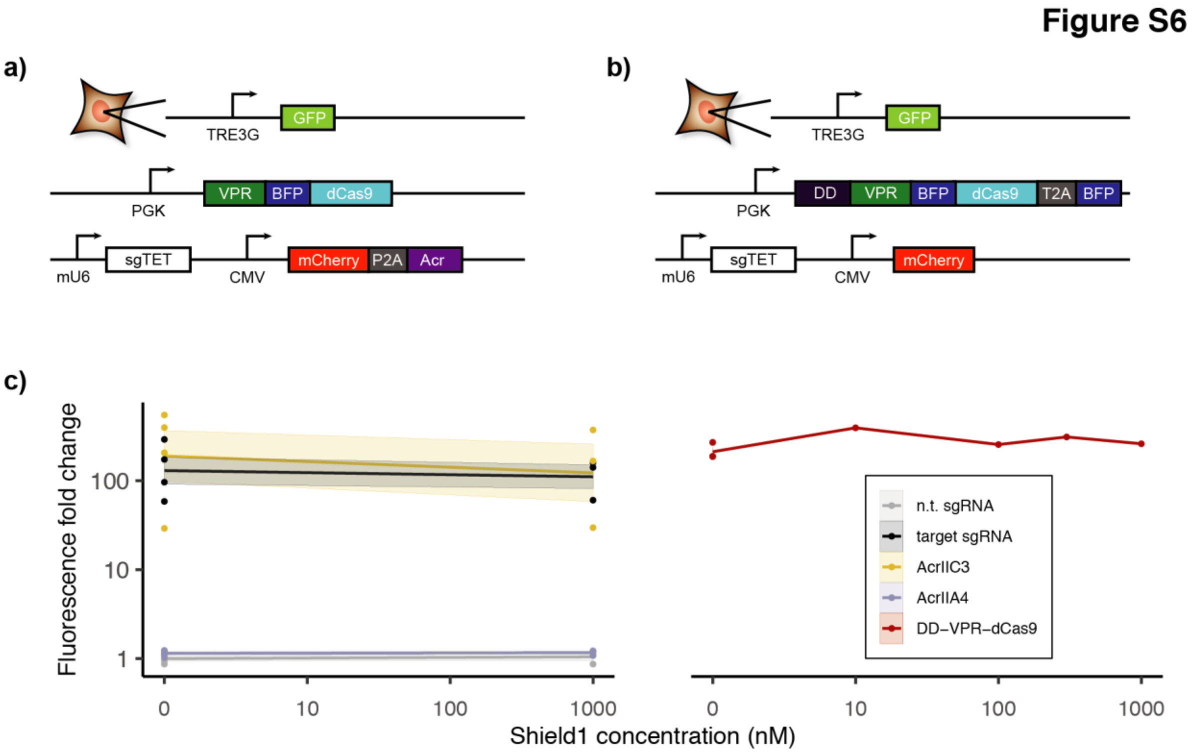

Supplementary Figure 6: effects of Shield1 on CRISPRa, Acr, and DD-VPR-dCas9

a) Control plasmids for CRISPRa, as well as CRISPRa with Acr lacking DD are transiently transfected into a reporter cell. **b**) A DD-domain is fused to VPR-dCas9 and assessed for CRISPRa activity. **c**) (left) Activity of CRISPRa with non-targeting guide (n.t. sgRNA), on-target guide (target sgRNA), on-target guide with AcrIIC3 (AcrIIC3) and on-target guide with AcrIIA4 in presence and absence of Shield1. (right) Activity of CRISPRa with DD-VPR-dCas9 fusion across Shield1 concentrations. Shaded regions indicate ± s.e.m. n=3 for all conditions except non-targeting sgRNA, target sgRNA, and AcrIIC3 0 nM conditions (n=4) and DD-VPR-dCas9 at Shield1 concentrations above 0 nM (n=1). Source data are provided as a Source Data file.

Figure S7

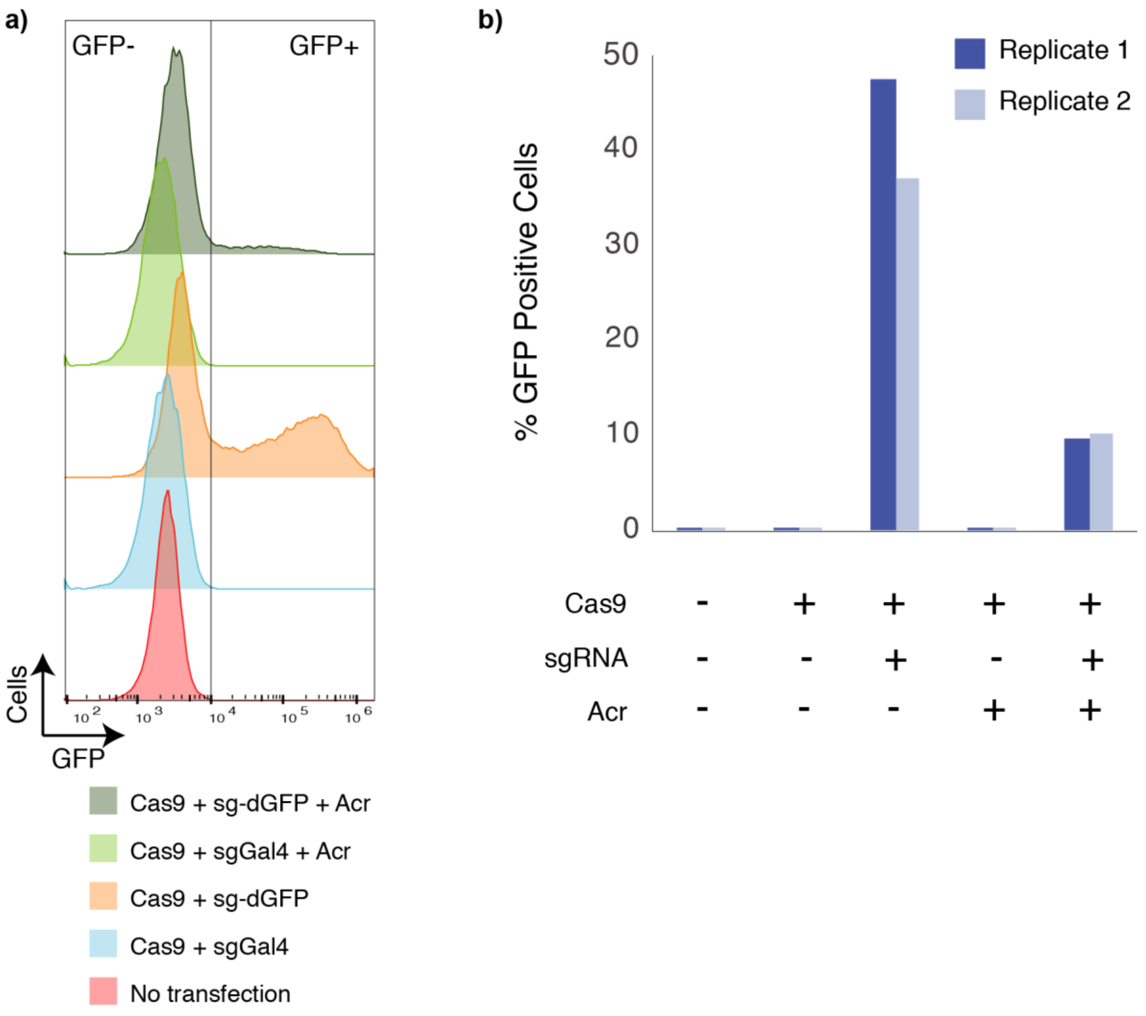

Supplementary Figure 7: AcrIIA4 mediates editing efficiency in a mammalian reporter system
A cell line was generated with an integrated editing reporter: a split GFP with the complementary strand in an out-of-frame context. Indels induced by Cas9 editing allow for the formation of in-frame expression, resulting in gain of fluorescence in the edited cell population. **a**) Flow cytometry traces demonstrating an increase in GFP expression upon genome editing, and diminished efficiency of editing in presence of AcrIIA4. **b**) Quantified proportion of GFP-positive cells in presence and absence of Acr for two experimental replicates. Source data are provided as a Source Data file.

Figure S8

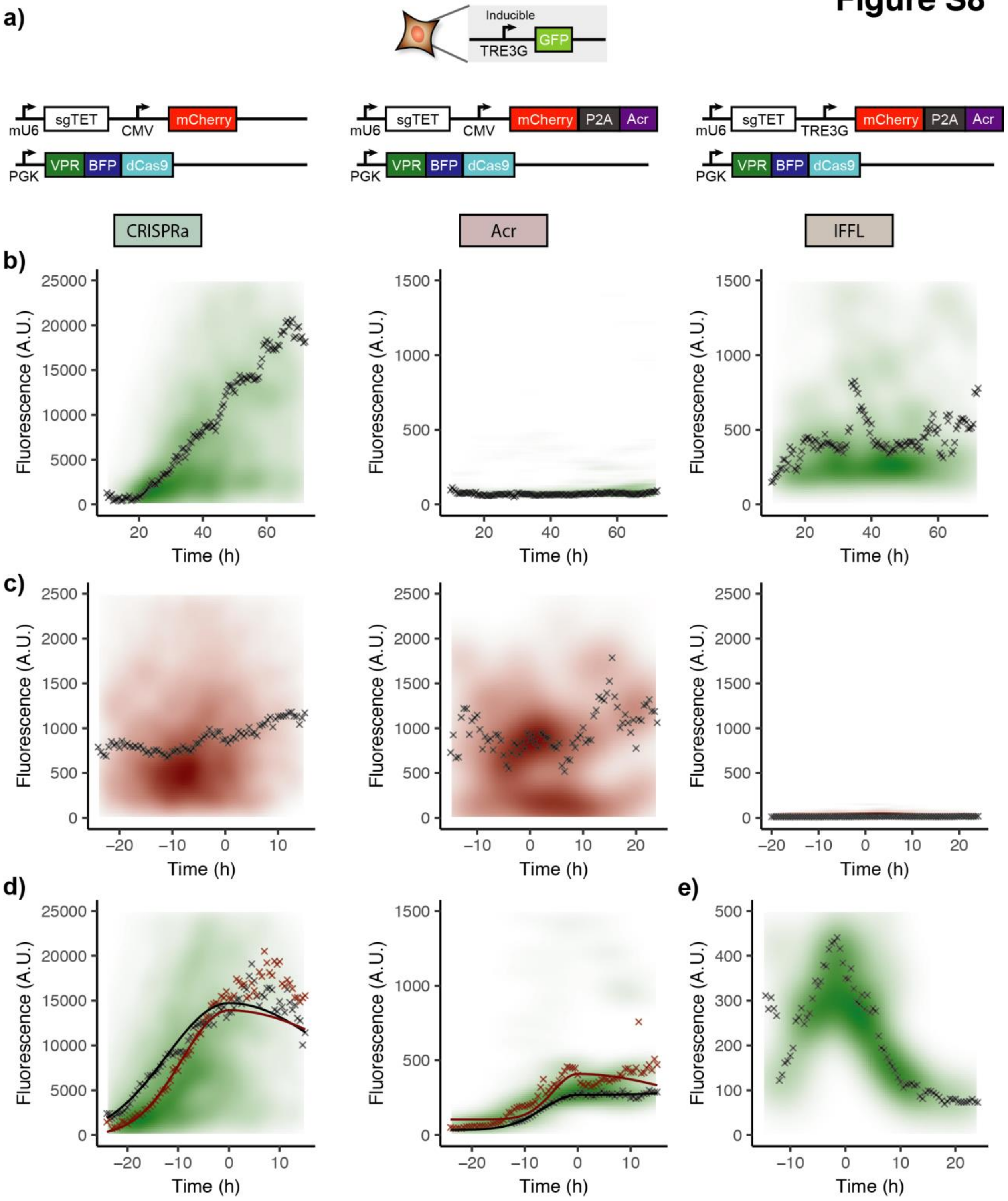

Supplementary Figure 8: circuit performance assessed by live-cell microscopy

a) Experimental scheme for live-cell microscopy assays: reporter cells with inducible GFP were stably integrated with plasmid with sgRNA and constitutive expression of mCherry alone (CRISPRa condition), constitutive expression of mCherry + AcrIIA4 (Acr condition), or inducible expression of mCherry + AcrIIA4 (IFFL condition). The circuits were started by transient

transfection of plasmid containing VPR-dCas9. **b)** Unaligned density plots (green) of computationally detected cell traces across CRISPRa (left), Acr (center), and IFFL (right) conditions. Median fluorescence is plotted as discrete points. Source data are provided as a Source Data file. **c)** Density plots of mCherry expression (red) with median fluorescence plotted as discrete points for CRISPRa (left), Acr (center), and IFFL (right) conditions. Data are aligned as and correspond to cells for GFP data shown in (**d**) and Figure 6c. Source data are provided as a Source Data file. **d)** Aligned density plots of two separate experiments (black and red) for CRISPRa (left) and Acr (right) conditions. Median fluorescence is plotted as discrete points, and median cell response fit is plotted as solid lines. Number of cell traces is n=472 for CRISPRa and n=6 for Acr conditions (only 6/97 cells passed alignment step due to low overall fluorescence). Source data are provided as a Source Data file. **e)** Aligned density plot and median fluorescence of transient transfection of both IFFL and VPR-dCas9 plasmids from n=8 cells (8/64 passed alignment step). Source data are provided as a Source Data file.

Figure S9

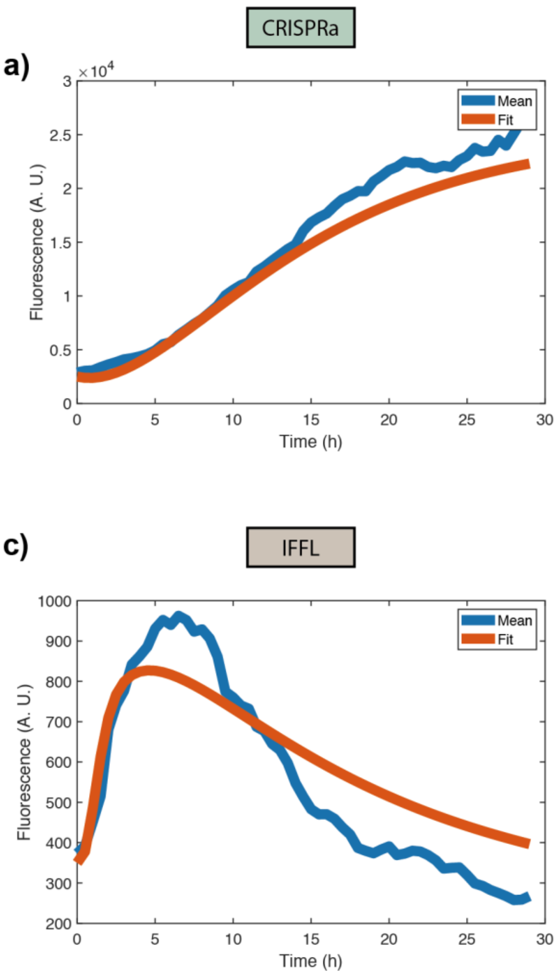

Supplementary Figure 9: Parameterization of model based on recapitulation of experimental circuit response

Comparison of mean fluorescence data of experimental traces and parameterized models (see Supplementary Modeling, below) for **a)** CRISPRa; **b)** Acr; and **c)** IFFL circuit conditions.

Supplementary Methods

Supplementary modeling
We construct a set of simple kinetic models involving production and degradation of relevant species for each circuit condition in an attempt to qualitatively recapitulate and describe the observed experimental behavior.

Model 1: CRISPRa condition

In this condition, VPR-dCas9 (*C*) is expressed from a constitutive promoter, and the reporter (*G*) is driven in a VPR-dCas9-dependent fashion. We make simplifying assumptions that sgRNA concentration is saturating and that production of GFP is proportional to VPR-dCas9 concentration. Further, we bunch all production processes in a single term. We therefore have the following rate equations governing this model:

$$\frac{dC}{dt} = k_1 - k_2 C$$

$$\frac{dG}{dt} = k_3 C - k_4 G$$

Model 2: IFFL condition

We now add an Acr species (*A*), with production also driven in a VPR-dCas9-dependent fashion, and assume that Acr may bind to dCas9, irreversibly inactivating it. This results in the following model:

$$\frac{dC}{dt} = k_1 - k_2 C - k_7 AC$$

$$\frac{dA}{dt} = k_5 C - k_6 A - k_7 AC$$

$$\frac{dG}{dt} = k_3 C - k_4 G$$

Model 3: Acr condition

As Model 2, except Acr is produced constitutively:

$$\frac{dC}{dt} = k_1 - k_2C - k_7AC$$

$$\frac{dA}{dt} = k_8 - k_6A - k_7AC$$

$$\frac{dG}{dt} = k_3C - k_4G$$

Because the Acr is stably integrated, we assume a steady-state initial concentration in our model to be given by

$$A_0 = \frac{k_8}{k_6}$$

Parameterization of models and sensitivity analysis

Solutions for these equations were generated numerically (MATLAB). We parameterized the models by fitting to aligned cell-tracking traces (Model 1 and Model 2) and unaligned traces (Model 3). In order to narrow the explored parameter space, we fit models to experimental data consecutively, propagating derived constants forward to subsequent models.

Based on the derived parameters from Model 2, we individually varied each of the 7 rate constants to generate computational predictions of circuit behavior in response to various perturbations in rate constants (shown in Figure 6).

Supplementary Table 1 – Cell lines

Cell line	Construct / Genotype	Reference
HEK293T	wild-type	Clontech
HEK293T	TRE3G → dscGFP	This study
HEK293T	SV40 → eGFP	(Gao, *et al.*, 2016)[1]
HEK293T	EF1α → rtTA; TRE3G → KRAB-dCas9-HA-NLS-NLS-P2A-sfGFP	This study
HEK293T	EF1α → rtTA; TRE3G → dCas9-HA-NLS-NLS-VPR-P2A-sfGFP	This study
hiPSC	EF1α → rtTA; TRE3G → dCas9-HA-NLS-NLS-VPR-P2A-sfGFP	This study
CEN.PK2-1D	*MATα ura3-52; trp1-289; leu2-3, 112; his3Δ1; MAL2-8C; SUC2*	(Entian and Kötter, 2007)[2]
yJZC10	*MATa leu2-3,112 trp1-1 can1-100 ura3-1 his3-11,15 HO::rtTA-HygB TRP1::pTETO7-Venus LEU2::pTDH3-dCas9-3XNLS HIS3::pAdh-MCP-VP64*	(Zalatan, *et al.*, 2014)[3]
yJZC14	*MATa leu2-3,112 trp1-1 can1-100 ura3-1 his3-11,15 HO::rtTA-HygB TRP1::pTETO7-Venus LEU2::pTDH3-dCas9-3XNLS mfa2::pTEF1-mCherry-kanR*	Unpublished
HEK293T	EF1α → mCherry-P2A-AcrIIA4	This study
HEK293T	mU6 → sgTET; CMV → mCherry	This study
HEK293T	mU6 → sgTET; CMV → mCherry-P2A-AcrIIA4	This study
HEK293T	mU6 → sgTET; TRE3G → mCherry-P2A-AcrIIA4	This study

Supplementary Table 2 – Cas9 plasmids

Construct	Reference
PGK → KRAB-BFP-dCas9-HA-NLSx2	(Gao, *et al.*, 2016)[1]
PGK → VPR-BFP-dCas9-HA-NLSx2	(Gao, *et al.*, 2016)[1]
pRNR2 → Cas9-NLS-His6	This study
mU6 → sgPD1-ko; TRE3G → NLS-Cas9-NLS-eGFP	This study
CMV → dCas9-HA-VPR-mCherry	(Kipniss, *et al.*, 2017)[4]
CMV → NES-ARRB2-NES-TCS-dCas9-HA-VPR-mCherry	(Kipniss, *et al.*, 2017)[4]
PGK → DD-VPR-BFP-dCas9-HA-NLSx2-T2A-BFP	This study
PGK → VPR-BFP-dCas9-HA-NLSx2-P2A-AcrIIA4	This study

Supplementary Table 3 – sgRNA sequences

sgRNA	Sequence
sgTET	GTACGTTCTCTATCACTGATAGTTTAAGAGCTATGCTGGAAACAGCATAGCAAGTTTAAATAAGGCTAGTCCGTTATCAACTTGAAAAAGTGGCACCGAGTCGGTGCTTTTTTT
sgSV40	GAAAGTCCCCAGGCTCCCCAGCGTTTAAGAGCTATGCTGGAAACAGCATAGCAAGTTTAAATAAGGCTAGTCCGTTATCAACTTGAAAAAGTGGCACCGAGTCGGTGCTTTTTTT
sgmRFP_RR1	GAACTTTCAGTTTAGCGGTCTGTTTAAGAGCTATGCTGGAAACAGCATAGCAAGTTTAAATAAGGCTAGTCCGTTATCAACTTGAAAAAGTGGCACCGAGTCGGTGCTTTTTTT
sgCXCR4-1a	GCCTCTGGGAGGTCCTGTCCGGCTCGTTTAAGAGCTATGCTGGAAACAGCATAGCAAGTTTAAATAAGGCTAGTCCGTTATCAACTTGAAAAAGTGGCACCGAGTCGGTGCTTTTTTT
sgCXCR4-3a	GCAGACGCGAGGAAGGAGGGCGCGTTTAAGAGCTATGCTGGAAACAGCATAGCAAGTTTAAATAAGGCTAGTCCGTTATCAACTTGAAAAAGTGGCACCGAGTCGGTGCTTTTTTT
sgCXCR4-20i	gcagaagcggccaggacattggGTTTAAGAGCTATGCTGGAAACAGCATAGCAAGTTTAAATAAGGCTAGTCCGTTATCAACTTGAAAAAGTGGCACCGAGTCGGTGCTTTTTTT
sgTRP1	GGCCGGCATGGTCCCAGCCTCCTCGCTGGCGCCGGCTGGGCAACACCTTCGGGTGGCGAATGGGACTTTccggatcaagattgtacgtaGTTTTAGAGCTAGAAATAGCAAGTTAAAATAAGGCTAGTCCGTTATCAACTTGAAAAAGTGGCACCGAGTCGGTGCTTTT
sgTETO-2x (wt + f6) MS2	ACTTTTCTCTATCACTGATAGTTTTAGAGCTAGAAATAGCAAGTTAAAATAAGGCTAGTCCGTTATCAACTTGAAAAAGTGGCACCGAGTCGGTGCGGGAGCACATGAGGATCACCCATGTGCGACTCCCACAGTCACTGGGGAGTCTTCCC
sgGAL4	gagcactgtcctccgaacgtGTTTAAGAGCTAAGCTGGAAACAGCATAGCAAGTTTAAATAAGGCTAGTCCGTTATCAACTTGAAAAAGTGGCACCGAGTCGGTGCTTTTTTT
sg-dGFP	GGTGGCGGATCAGAAGGAGGgtttaagagctatgctggaaacagcatagcaagtttaaataaggctagtccgttatcaacttgaaaaagtggcaccgagtcggtgcttttttt
sgPD1-ko	ggaagcggcagtcctggcgtttAagagctaagctggaaacagcatagcaagttTaaataaggctagtccgttatcaacttgaaaaagtggcaccgagtcggtgcttttttt
sgRELA	gatctccacataggggccag
sgCDC42BPB	gagccgcaccttggccgaca

sgRNA sequences used in experiments: spacer sequence; HDV ribozyme, MS2.

For reporter experiments: sgSV40 was used as the negative control sgRNA for the TRE3G promoter, and sgTET was used as the negative control sgRNA for the SV40 promoter.

For CXCR4 experiments: sgCXCR4-1a and sgCXCR4-3a were used in combination for HEK293T activation; sgCXCR4-20i was used for repression; sgCXCR4-3a was used for hiPSC activation; sgmRFP_RR1 was used as the negative control sgRNA.

For gene editing reporter experiments, sgGAL4 was used as non-targeting guide and sg-dGFP for on-target guide. sgPD1-ko as above was used for WPC gene knock-out plasmid transfections. sgPD1-ko, sgRELA, and sgCDC42BPB spacer sequences were used with a modified scaffold sequence[5] for RNP delivery.

Supplementary Table 4 – Acr sequences

Protein domain	Sequence
AcrIIC1	MAKEVFKLKPELVTYKGCGWALACIKDGEIIDLTYVRDLGIEEYDENFDGLEPEIIYYDVVASQACKEVAYRYEEMGEFTFGLCSCWEFNVM
AcrIIC3	MAFKRAIIFTSFNGFEKVSRTEKRRLAKIINARVSIIDEYLRAKDTNASLDGQYRAFLFNDESPAMTEFLAKLKAFAESCTGISIDAWEIEESEYVRLPVERRDFLAAANGKEIFKI
AcrIIA1	MTIKLLDEFLKKHDLTRYQLSKLTGISQNTLKDQNEKPLNKYTVSILRSLSLISGLSVSDVLFELEDIEKNSDDLAGFKHLLDKYKLSFPAQEFELYCLIKEFESANIEVLPFTFNRFENEEHVNIKKDVCKALENAITVLKEKKNELL
AcrIIA2	MTLTRAQKKYAEAMHEFINMVDDFEESTPDFAKEVLHDSDYVVITKNEKYAVALCSLSTDECEYDTNLYLDEKLVDYSTVDVNGVTYYINIVETNDIDDLEIATDEDEMKSGNQEIILKSELK
AcrIIA3	MTKYNKSEIMKNAWAMFNSYEWDVENFKFVSAENKTFSNCLKEAWAEEKEYVERKAKETAEAPRSEEAKAWDWACRKLNVNDLQNIDATDKVFYVVDMQKEMWTSNVWAQAIKAVELYVKLGLA
AcrIIA4	MNINDLIREIKNKDYTVKLSGTDSNSITQLIIRVNNDGNEYVISESENESIVEKFISAFKNGWNQEYEDEEEFYNDMQTITLKSELN
AcrIIA5 v1	MAYGKSRYNSYRKRNFSISDNQRREYAKKMKELEQAFENLDGWYLSSMKDSAYKDFGKYEIRLSNHSADNRYHDLENGRLIVNVKASKLNFVDIIENKLGKIIEKIDTLDLDKYRFINATKLERDIKCYYKGYKTKKDVI
AcrIIA5 v2	MAYGKSRYNSYRKRSFNRSNKQRREYAQEMDRLEKAFENLDGWYLSSMKDSAYKDFGKYEIRLSNHSADNKYHDLENGRLIVNIKASKLNFVDIIENKLDKIIEKIDKLDLDKYRFINATNLEHDIKCYYKGFKTKKEVI
AcrIIA6	MKINDDIKELILEYMSRYFKFENDFYKLPGIKFTDANWQKFKNGGTDIEKMGAARVNAMLSCLFEDFELAMIGKAQTNYYIDNSLKLNMPFYAYYDMFKKQLLINWLKNNRDDVICGTGRMYTASGNYIANAYLEVALESSRLGGGEYMLQMRFKNYSRSQEPIPSGRQNRLEWIENNLENIR

Supplementary References

1. Gao, Y. *et al.* Complex transcriptional modulation with orthogonal and inducible dCas9 regulators. *Nat. Methods* **13,** 1043–1049 (2016).
2. Entian, K.-D. & Kötter, P. in *Methods in Microbiology* **36,** 629–666 (Academic Press, 2007).
3. Zalatan, J. G. G. *et al.* Engineering Complex Synthetic Transcriptional Programs with CRISPR RNA Scaffolds. *Cell* **160,** 339–350 (2014).
4. Kipniss, N. H. *et al.* Engineering cell sensing and responses using a GPCR-coupled CRISPR-Cas system. *Nat. Commun.* **8,** 2212 (2017).
5. Hendel, A. *et al.* Chemically modified guide RNAs enhance CRISPR-Cas genome editing in human primary cells. *Nat. Biotechnol.* **33,** 985–989 (2015).
