# Supplementary material for: Anti-CRISPR-mediated control of gene editing and synthetic circuits in eukaryotic cells
Source: Nat Commun. 2019 Jan 14;10:194. doi: 10.1038/s41467-018-08158-x (PMC6331597; doi:10.1038/s41467-018-08158-x)
Supplement: Supplementary file 2 — Description of Additional Supplementary Files [file 41467_2018_8158_MOESM2_ESM.pdf]

Description of Additional Supplementary Files

File Name: Supplementary Movie 1
Description: CRISPRa condition (unperturbed GFP activation).

File Name: Supplementary Movie 2
Description: Acr condition (constitutive expression of Acr inhibits GFP activation).

File Name: Supplementary Movie 3
Description: IFFL condition (induced activation of GFP and Acr). Selected cells demonstrating pulsatile reporter expression are manually annotated.

File name: Supplementary Movie 4
Description: Computationally selected cell-tracking traces of IFFL condition generated from image analysis pipeline.
