# Supplementary material for: Anti-CRISPR-mediated control of gene editing and synthetic circuits in eukaryotic cells
Source: Nat Commun. 2019 Jan 14;10:194. doi: 10.1038/s41467-018-08158-x (PMC6331597; doi:10.1038/s41467-018-08158-x)
Supplement: Supplementary file 7 — Reporting Summary [file 41467_2018_8158_MOESM7_ESM.pdf]

nature research

Corresponding author(s): Lei S. Qi

Last updated by author(s): Dec 13, 2018

Reporting Summary

Nature Research wishes to improve the reproducibility of the work that we publish. This form provides structure for consistency and transparency in reporting. For further information on Nature Research policies, see Authors & Referees and the Editorial Policy Checklist.

Statistics

For all statistical analyses, confirm that the following items are present in the figure legend, table legend, main text, or Methods section.

n/a	Confirmed	
☐	☒	The exact sample size (n) for each experimental group/condition, given as a discrete number and unit of measurement
☐	☒	A statement on whether measurements were taken from distinct samples or whether the same sample was measured repeatedly
☐	☒	The statistical test(s) used AND whether they are one- or two-sided<br>*Only common tests should be described solely by name; describe more complex techniques in the Methods section.*
☒	☐	A description of all covariates tested
☒	☐	A description of any assumptions or corrections, such as tests of normality and adjustment for multiple comparisons
☐	☒	A full description of the statistical parameters including central tendency (e.g. means) or other basic estimates (e.g. regression coefficient) AND variation (e.g. standard deviation) or associated estimates of uncertainty (e.g. confidence intervals)
☐	☒	For null hypothesis testing, the test statistic (e.g. F, t, r) with confidence intervals, effect sizes, degrees of freedom and P value noted<br>*Give P values as exact values whenever suitable.*
☒	☐	For Bayesian analysis, information on the choice of priors and Markov chain Monte Carlo settings
☒	☐	For hierarchical and complex designs, identification of the appropriate level for tests and full reporting of outcomes
☒	☐	Estimates of effect sizes (e.g. Cohen's d, Pearson's r), indicating how they were calculated

Our web collection on statistics for biologists contains articles on many of the points above.

Software and code

Policy information about availability of computer code

Data collection	Associated bundled software for acquisition of data using flow cytometry and live cell microscopy.
Data analysis	FlowJo for flow cytometry analysis. FIJI, and custom ImageJ, Python, and R scripts for data handling and analysis.

For manuscripts utilizing custom algorithms or software that are central to the research but not yet described in published literature, software must be made available to editors/reviewers. We strongly encourage code deposition in a community repository (e.g. GitHub). See the Nature Research guidelines for submitting code & software for further information.

Data

Policy information about availability of data

All manuscripts must include a data availability statement. This statement should provide the following information, where applicable:

- Accession codes, unique identifiers, or web links for publicly available datasets
- A list of figures that have associated raw data
- A description of any restrictions on data availability

The data and code that support the findings of this study are available upon request to the corresponding author (L.S.Q.).

Field-specific reporting

Please select the one below that is the best fit for your research. If you are not sure, read the appropriate sections before making your selection.

☒ Life sciences ☐ Behavioural & social sciences ☐ Ecological, evolutionary & environmental sciences

For a reference copy of the document with all sections, see nature.com/documents/nr-reporting-summary-flat.pdf

Life sciences study design

All studies must disclose on these points even when the disclosure is negative.

Sample size	Sample sizes were not pre-selected. Target sizes of duplicate or triplicate were pursued to ascertain internal consistency, with additional replicates done as part of larger experiments. Data are mostly qualitative in nature, and sample sizes seem sufficient to illustrate the large effects seen.
Data exclusions	No data exclusions.
Replication	Replications were successful.
Randomization	Allocation was not random. We expect that covariates should be minimal, since these are cell assays using standard cell culture materials.
Blinding	Blinding was not performed. We endeavored to treat all samples via the same analysis pipeline to minimize potential bias. Since data collection and analysis was driven computationally, we believe blinding to be of minor importance for our research.

Reporting for specific materials, systems and methods

We require information from authors about some types of materials, experimental systems and methods used in many studies. Here, indicate whether each material, system or method listed is relevant to your study. If you are not sure if a list item applies to your research, read the appropriate section before selecting a response.

Materials & experimental systems

n/a	Involved in the study	
☐	☒	Antibodies
☐	☒	Eukaryotic cell lines
☒	☐	Palaeontology
☒	☐	Animals and other organisms
☒	☐	Human research participants
☒	☐	Clinical data

Methods

n/a	Involved in the study	
☒	☐	ChIP-seq
☐	☒	Flow cytometry
☒	☐	MRI-based neuroimaging

Antibodies

Antibodies used	APC-labeled anti-CXCR4 (BioLegend #306510)
Validation	The antibody undergoes standard QC testing by the provider. Used often in the lab, including in previously published studies (such as Gao, et al., 2016).

Eukaryotic cell lines

Policy information about cell lines

Cell line source(s)	HEK293T: Clontech; hiPSC: from collaborators.
Authentication	None.
Mycoplasma contamination	Not tested.
Commonly misidentified lines (See ICLAC register)	None.

Flow Cytometry

Plots

Confirm that:

☐ The axis labels state the marker and fluorochrome used (e.g. CD4-FITC).

☒ The axis scales are clearly visible. Include numbers along axes only for bottom left plot of group (a 'group' is an analysis of identical markers).

☐ All plots are contour plots with outliers or pseudocolor plots.

☐ A numerical value for number of cells or percentage (with statistics) is provided.

Methodology

Sample preparation	Standard procedures: detachment of cells from tissue culture plates via trypsin / Accutase, washing into DPBS + 10 % FBS. Immunostaining was performed in this buffer, as necessary, according to the manufacturer's protocol.
Instrument	Beckman Coulter CytoFLEX
Software	Collection: standard software. Preliminary analysis: FlowJo. Final analysis: custom scripts.
Cell population abundance	Ranges depending on experiment from ~5 % to majority of cells (> 50 %).
Gating strategy	FSC / SSC determined by bulk population and comparison to other cells. Singlet gate, followed by appropriate fluorescent gates (detection of plasmid presence, if available).

☐ Tick this box to confirm that a figure exemplifying the gating strategy is provided in the Supplementary Information.
